# Comparison of the pharmacokinetic variations of different concentrations of ropivacaine used for serratus anterior plane block in patients undergoing thoracoscopic lobectomy: a population pharmacokinetics analysis

**DOI:** 10.3389/fphar.2025.1540606

**Published:** 2025-03-26

**Authors:** Jing Ling, Caomei Xu, Lingkai Tang, Lan Qiu, Nan Hu

**Affiliations:** ^1^ Department of Pharmacy, The First People’s Hospital of Changzhou/The Third Affiliated Hospital of Soochow University, Changzhou, China; ^2^ Department of Anesthesiology, The First People’s Hospital of Changzhou/The Third Affiliated Hospital of Soochow University, Changzhou, China

**Keywords:** ropivacaine, serratus anterior plane block (SAPB), nonlinear mixed-effects model, population pharmacokinetics, thoracoscopic lobectomy

## Abstract

**Objective:**

Ropivacaine serratus anterior plane block is widely used in clinical analgesia in patients undergoing thoracoscopic surgery. Different concentrations of ropivacaine have different analgesic effects, and the safety is highly correlated with the plasma concentration. In this study, the nonlinear mixed effects modeling (NONMEM) method was used to investigate the population pharmacokinetics (PPK) characteristics of ropivacaine and explored the relationship between the covariates on the pharmacokinetic parameters of ropivacaine, in order to provide a theoretical basis for the rational use of ropivacaine.

**Methods:**

This study was approved by the Ethics Committee of the First People’s Hospital of Changzhou. The informed consent of patients was obtained. A total of 43 patients who underwent thoracoscopic pneumonectomy in our hospital from April to December 2023 were included. Patients were randomly assigned to four ropivacaine concentration groups of 0.25%, 0.375%, 0.5%, and 0.75%, respectively, and administered with a dose of 3 mg/kg. Arterial blood was taken at 1, 15, 30, 45 min, 1, 2, 4, 8, 12, and 24 h after ropivacaine administration through superficial serratus anterior plane block. The concentration of ropivacaine was detected by liquid chromatography-tandem mass spectrometry (LC-MS/MS). The PPK model was constructed by NONMEM. The final model was verified by using the goodness of fit, visual predictive check (VPC) and normalized predictive distribution error (NPDE). Monte Carlo simulation was applied to evaluate and optimize the dosing regimens.

**Results:**

A total of 388 plasma concentration data from 41 patients were used to establish the model. Eighteen blood concentrations from the other two patients were used for external validation. A two-compartment with zero-order and first-order mixed absorption model was the best model. The proportion of zero-order absorption was 27.4%, the absorption time of zero-order absorption was 0.49 h and the zero-order absorption infusion time was 0.015 h. The first-order absorption rate constant (ka)was correlated with the concentration of ropivacaine. The ka of ropivacaine were 32.0, 19.4 and 14.4 h^−1^ for 0.25%, 0.5%, and 0.75% ropivacaine, respectively, which indicating that the peak time (T_max_) of low-concentration ropivacaine was significantly shortened. Other pharmacokinetic parameters results were as follows: CL/F(L/h) = 7.475, Vc/F(L) = 125, Q/F(L/h) = 14.7, Vp/F(L) = 197. In addition, the platelet count has an effect on the Vc/F. The simulation results demonstrate the total dose of ropivacaine is recommended not to exceed 300 mg to avoid the occurrence of adverse reactions.

**Conclusion:**

This is the first population pharmacokinetic study of ropivacaine superficial serratus anterior plane block in patients undergoing thoracoscopic pulmonary resection. The model exhibits excellent stability and reliability, thereby offering valuable insights into personalized clinical drug administration. The concentration of ropivacaine and platelet count have significant impacts on the pharmacokinetic parameters of ropivacaine.

## Introduction

The serratus anterior plane block is a blockade technique that has been widely applied in clinical practice in recent years (Blanco et al., 2013). Local anesthetics are injected into the superficial or deep spaces of the serratus anterior muscle. The drugs gradually infiltrate along the lateral side of the intercostal nerves without exerting a direct blocking effect on the intercostal nerves, ultimately achieving analgesia on the anterolateral chest wall (Finnerty et al., 2020). It has been used in breast surgeries, rib fractures, and thoracotomy to manage pain of the anterolateral chest wall (Xie et al., 2021).

Ropivacaine is a long-acting amide-type local anesthetic with both anesthetic and analgesic effects. It exhibits linear pharmacokinetic characteristics, with maximum plasma concentration directly proportional to the dose, and safety being highly correlated with the blood drug concentration. The recommended dosage range for regional block at different sites in the instruction manual is wide. The selection of ropivacaine concentration and dose in serratus anterior plane block is mainly based on the clinical experience of anesthesiologists, and there is no uniform standard for the most appropriate concentration and dose. The commonly used concentration of ropivacaine is 0.25%–0.75% with the volume of 20–40 mL. In this study, we used 3 mg/kg of ropivacaine, which is the maximum dose recommended by some authors (Rosenberg et al., 2004). Occasionally, in order to obtain a longer duration of analgesia, a higher concentration of local anesthetics has been considered to apply clinically. However, the incidence of local anesthetic systemic toxicity may increase with the dose increase (Satsumae et al., 2008). When the peak arterial drug concentration reaches 4.3 (3.4–5.3) μg/mL, the probability of neurotoxic or cardiotoxic reactions in patients increases (Knudsen et al., 1997). Additionally, different concentrations of ropivacaine have different analgesic effects after serratus anterior plane block (Huang et al., 2020). A previous study believed that 0.5% ropivacaine nerve block and analgesia is good, but it is easy to cause the incidence of muscle tremor, respiratory depression and other adverse reactions, which may be related to the excessive concentration of ropivacaine (Zhu et al., 2020). In consideration of the pharmacokinetics of ropivacaine is closely related to its safety (Riff et al., 2022). It is necessary to identify the pharmacokinetic differences between various concentrations of ropivacaine.

There have been fewer reports on the population pharmacokinetic studies of ropivacaine (Matsota et al., 2024; Riff et al., 2018; Schwenk et al., 2023), but none were published in patients with serratus anterior plane block. Therefore, it is crucial to establish a population pharmacokinetic model for analyzing the pharmacokinetic characteristics of different concentrations of ropivacaine on the patients with serratus anterior plane block and quantitatively evaluating the impact of covariates on the pharmacokinetic parameters.

Thus, the aims of this study were to describe the population pharmacokinetic characteristics of ropivacaine for the serratus anterior plane block in patients undergoing video-assisted thoracoscopic lobectomy and to provide theoretical support for the rational use of ropivacaine in clinical practice.

## Materials and methods

### Patients

Patients who underwent thoracoscopic lung resection at the First People’s Hospital of Changzhou from April 2023 to August 2023 were included. They received ropivacaine for anterior serratus plane block with concentrations of 0.25%, 0.375%, 0.5%, and 0.75%, respectively, at a dose of 3 mg/kg. Arterial blood was drawn at 1, 15, 30, 45 min, 1, 2, 4, 8, 12, and 24 h after the block was completed. The inclusion criteria were as follows: (1) Understanding the research protocol and signing the informed consent form; (2) Undergoing primary elective thoracoscopic surgery; (3) American Society of Anesthesiologists (ASA) grades I–III; (4) No chronic painful diseases or cognitive dysfunction were identified before the operation; (5) No obvious organ dysfunction. Exclusion criteria: (1) Having peripheral neuropathy or injury; (2) recent use of anesthetic drugs or allergy to anesthetic drugs; (3) block failure; (4) internal environment disorder or severe hemodynamic instability during the perioperative period. It is considered that the peak time of ropivacaine is faster, and the peak concentration is higher when using the deep serratus anterior plane block (Griffiths et al., 2010; He et al., 2023; Rahiri et al., 2017). Therefore, we selected the superficial serratus anterior plane block.

This study was approved by the ethics committee, and informed consent was obtained from the subjects before the study.

### Determination of ropivacaine concentration

Take 50 μL of plasma, add 5 μL of internal standard diazepam (5 μg/mL), then add 200 μL of methanol. Vortex and shake for 3 min, and centrifuge at 16,400 r/min for 10 min. Take 50 μL of the supernatant, add 450 μL of pure water for dilution, centrifuge at 16,400 r/min for 10 min. The supernatant was used for the determination of the blood drug concentration of ropivacaine by liquid chromatography-tandem mass spectrometry (LC-MS/MS).

Chromatographic column: Phenomenex Kinetex_C18; Mobile phase A is an aqueous solution composed of 0.01% formic acid and 10 mmol L^−1^ ammonium acetate, while mobile phase B is a methanol solution containing 0.01% formic acid. Gradient elution (A:B): 0–0.5 min, 30% B; 0.5–2.5 min, from 30% B to 100% B; 2.5–4.0 min, 100% B; 4.0–4.5 min, from 100% B to 30% B; 4.5–5.0 min, 30% B. Flow rate: 0.4 mL min^−1^; Injector temperature: 4°C; Column temperature: 40°C; Injection volume: 2 μL. Electro-Spray Ionization (ESI) source, positive ion Multiple Reaction Monitoring (MRM) mode. The quantitative ion pair for ropivacaine is m/z → 275.3/126.1. The regression equation of the standard curve of ropivacaine in plasma by this method was: Y = 13.02681X + 0.00690, *r* ^2^ = 0.9992. The lowest limit of quantification was 0.004 μg mL^−1^. The intra-batch and inter-batch precisions were 5%–10%. The accuracy was 89.31%–111.07%. The extraction recovery rate was 85.31%–98.67%.

### Population pharmacokinetic modeling

The nonlinear mixed effects model (NONMEM) was used to establish the population pharmacokinetic (PPK) model. The estimation method was the first-order conditional estimation algorithm with interaction (FOCEI).

The establishment of the structural model was conducted by fitting with absorption models such as zero-order absorption, first-order absorption, mixed zero-order and first-order absorption, zero-order absorption with delay, first-order absorption with delay, and progressive absorption (Transit) model. The investigation of the compartment model employed one-compartment model, two-compartment model, and three-compartment model.

Random effects include inter- individual variation and residual variation. The inter-individual variability in PK parameters was described with an exponential model:

$$P_{j}=\hat{P}\times \exp\left(\eta_{j}\right)$$

where $P_{j}$ represents pharmacokinetic parameter estimation for the *j*th individual, $\hat{P}$ represents the population typical value of the parameters, and $\eta_{j}$ is a random variable distributed with a mean of zero and variance of ω^2^. Residual variability was evaluated using proportional and additive combined error model:

$$C_{ij}={\hat{C}}_{ij}\times \left(1+\varepsilon_{1}\right)+\varepsilon_{2}$$

where $C_{ij}$ represents the *j*th observation for the *i*th patient, ${\text{and}\;\hat{C}}_{ij}$ represents the *j*th predicted value for the *i*th patient. $\varepsilon_{1}$ and $\varepsilon_{2}$ are the intra-individual variability with a mean of zero and variance of σ_1_^2^ and σ_2_^2^, respectively.

The covariates were screened using the forward inclusion and backward elimination methods. It was considered significant when the inclusion of a covariate decreased the objective unction value (OFV) by at least 3.84 (P < 0.05) and increased the OFV by at least 6.63 (P < 0.01) in the backward step. The correlations between the covariates and pharmacokinetic parameters were explored by the linear plots and box plots. Age, sex, weight, the concentration of ropivacaine, white blood cell, red blood cells, hemoglobin, hematocrit, platelets, albumin, total bilirubin, alkaline phosphatase, alanine transaminase, aspartate transaminase, serum creatinine, serum total bile acid, glomerular filtration rate, uric acid and co-administered drugs, etc., were tested by forward and backward selection to determine if these potential covariates affected the ropivacaine pharmacokinetic parameters. Only comedications >5% in all patients were tested.

### Model validation

The model was evaluated using goodness of fit plots, visual predictive check (VPC), and normalized predictive distribution error (NPDE). Furthermore, an independent dataset is used for external verification.

### Simulations

Monte Carlo simulation was performed for each dosage regimen based on the final model. According to the simulation results, the distribution of peak concentrations is estimated and compared with the threshold values of toxic effects.

### Statistical analysis

Statistical analysis was performed using IBM SPSS (version 16.0). Categorical data were compared using the chi-square test. Continuous data with normal distribution were analyzed by one-way analysis of variance (ANOVA), and with non-normal distribution were analyzed by Kruskal-Wallis test. Winnonlin was used to calculate the pharmacokinetic parameters of ropivacaine in the three groups. P-values less than 0.05 were considered statistically significant.

## Results

### Patient characteristics

A total of 43 patients were included in the study. The number of patients with concentrations of ropivacaine at 0.25%, 0.375%, 0.5%, and 0.75% were recorded as 12, 2, 14, and 15 respectively. Among them, forty-one patients were from a randomized double-blind trial. The patients were divided into 0.25%/0.5%/0.75% groups by random number table method. A total of 388 plasma concentration data from these forty-one patients were used to establish the population pharmacokinetic models. The general data is presented in Table 1. The results of the pharmacokinetic parameters are shown in Table 2. Eighteen blood concentrations of two patients with 0.375% concentration were used for external validation of the population pharmacokinetic model.

**TABLE 1:** The demographics and clinical information of patients.

Characteristic	The concentration of ropivacaine			
	0.25% (n = 12)	0.5% (n = 14)	0.75% (n = 15)	*p*
Age/y	60.5 (31–75)	58 (33–74)	59 (47–68)	0.718
Weight/kg	57.3 (50–71)	60.5 (50–73)	60 (50–81)	0.381
White blood cell count (×10^9^ L^–1^)	5.26 (3.34–9.26)	6.97 (3.26–13.16)	6.31 (3.82–10.69)	0.247
Red blood cell count (×10^9^ L^–1^)	4.09 (3.43–5.11)	4.33 (3.45–4.74)	4.17 (3.57–5.5)	0.181
Platelet count (×10^9^ L^–1^)	182 (43–341)	213 (133–344)	187 (132–240)	0.208
Alanine aminotransferase (U·L^–1^)	15.3 (6.5–30.1)	16.6 (9.6–202.6)	20.1 (9.8–47.8)	0.410
Aspartate aminotransferase (U·L^–1^)	20.6 (18.6–34)	20.9 (13.2–189.0)	24.4 (16.7–41.7)	0.607
Serum creatinine (mmol/L)	65 (49–76)	57.5 (43–76)	65 (53–97)	0.409
Combination drugs				
Propofol (%)	25.0	71.4	66.7	0.035*
Dyclonine mucilage (%)	58.3	42.8	93.3	0.013*
Lidocaine (%)	58.3	57.1	53.3	0.962

Results for continuous covariates are presented as median (range), and results for categorical covariates are presented as percentage. *P < 0.05.

**TABLE 2:** Comparison of the pharmacokinetic parameters of ropivacaine in three groups of patients.

Parameter	The concentration of ropivacaine			*P*
	0.25% (n = 12)	0.5% (n = 14)	0.75% (n = 15)	
Dose/mg	178.7 ± 20.1	185.5 ± 20.6	191.1 ± 28.3	0.445
T_max_/h	0.5 (0.5, 2)	0.75 (0.25, 2)	1.0 (0.5, 4)	0.245
C_max_/(ng/mL)	1,249.0 ± 429.3	1,498.4 ± 456.1	1,660.3 ± 408.0	0.037
AUC_0-t_/(ng⋅h/mL)	10,853.7 ± 3,369.4	11,391.4 ± 3,932.0	11,872.0 ± 5,668.6	0.936

### Population pharmacokinetic analysis

**FIGURE 1:**
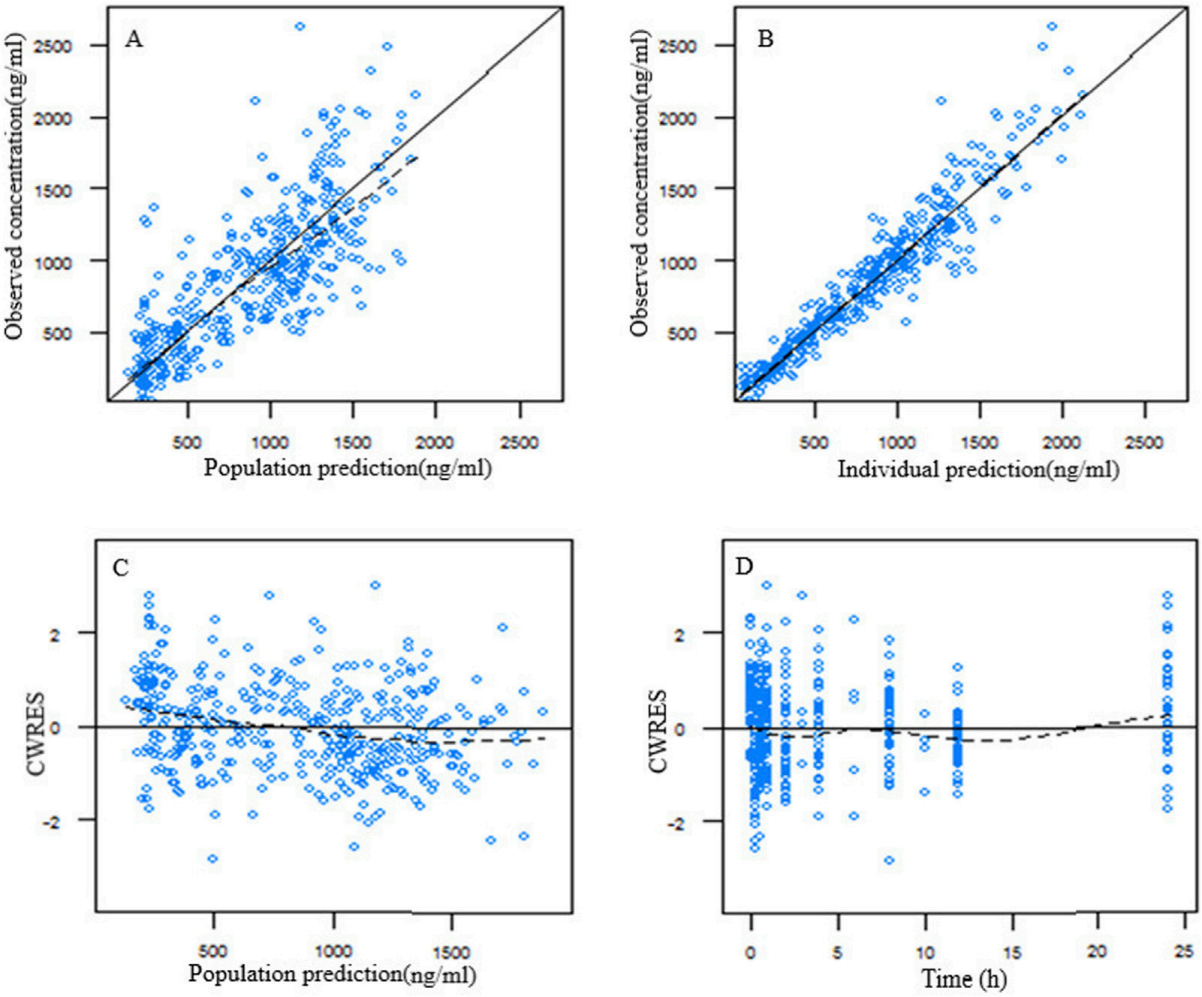
Goodness of fit plot for the final population pharmacokinetic model of ropivacaine. **(A)** Population predicted vs. observed concentration; **(B)** individual predicted vs. observed concentration; **(C)** conditional weighted residuals (CWRES) vs. population predicted concentration; **(D)** CWRES vs. time after dose.

A two-compartment incorporating both zero-order and first-order absorption kinetics was found to be the best base model due to lowest OFV and the best goodness of fit plots. All covariates were screened on the pharmacokinetic parameters of ropivacaine by the stepwise analysis method. Covariate screening results indicated that the concentration of ropivacaine significantly influenced the first-order absorption rate constant (ka), while platelet count affected the central compartment distribution volume (Vc/F). The parameters from the population pharmacokinetic fitting are presented in Table 3. The goodness of fit plot for the final model is illustrated in Figure 1. It is evident that the population predicted values are predominantly aligned with the observed values, and individual predicted values exhibit a uniform distribution around both sides of the identity line (y = x), demonstrating close clustering. Additionally, conditionally weighted residuals (CWRES) are evenly distributed about y = 0 and show no significant correlation with time or population predicted values, suggesting robust model fitting outcomes.

**TABLE 3:** Population pharmacokinetic parameter estimates results for ropivacaine.

Parameter	Parameter description	Estimate (RSE%)
F1 (%)	Fraction of drug absorption through the first-order absorption kinetics	72.6 (8.0%)
ALAG2 (h)	Absorption time lag of the zero-order absorption kinetics	0.49 (0.4%)
D2 (h)	The duration of administration for the zero-order absorption	0.015 (12.5%)
ka (h^-1^)	First-order absorption rate constant	
0.25% ropivacaine		32.0 (22.1%)
0.50% ropivacaine		19.4 (19.9%)
0.75% ropivacaine		14.4 (18.3%)
k (h^-1^)	First-order elimination rate constant	0.0598 (13.4%)
Vc/F (L)	Apparent distribution volume of the central compartment	125 (4.8%)
Q/F (L/h)	Apparent intercompartmental clearance	14.7 (31.5%)
Vp/F (L)	Apparent distribution volume of the peripheral compartment	197 (10.5%)
θ_PLT-Vc/F_ (%)	Effect of platelet count onV_C_/F	−0.438 (29.7%)
ωF_1_ (%)	Inter-individual variability of F_1_	21.1 (33.4%)
ωka (%)	Inter-individual variability of ka	58.2 (36.0%)
ωk (%)	Inter-individual variability of k	68.3 (23.3%)
ωVc/F (%)	Inter-individual variability of Vc/F	24.0 (27.2%)
ωVp/F (%)	Inter-individual variability of Vp/F	105.4 (29.2%)
δ_prop_ (%)	Proportional residuals	14.5 (21.7%)
δ_add_ (ng/mL)	Additional residuals	80.1 (30.8%)

### Model validation

**FIGURE 2:**
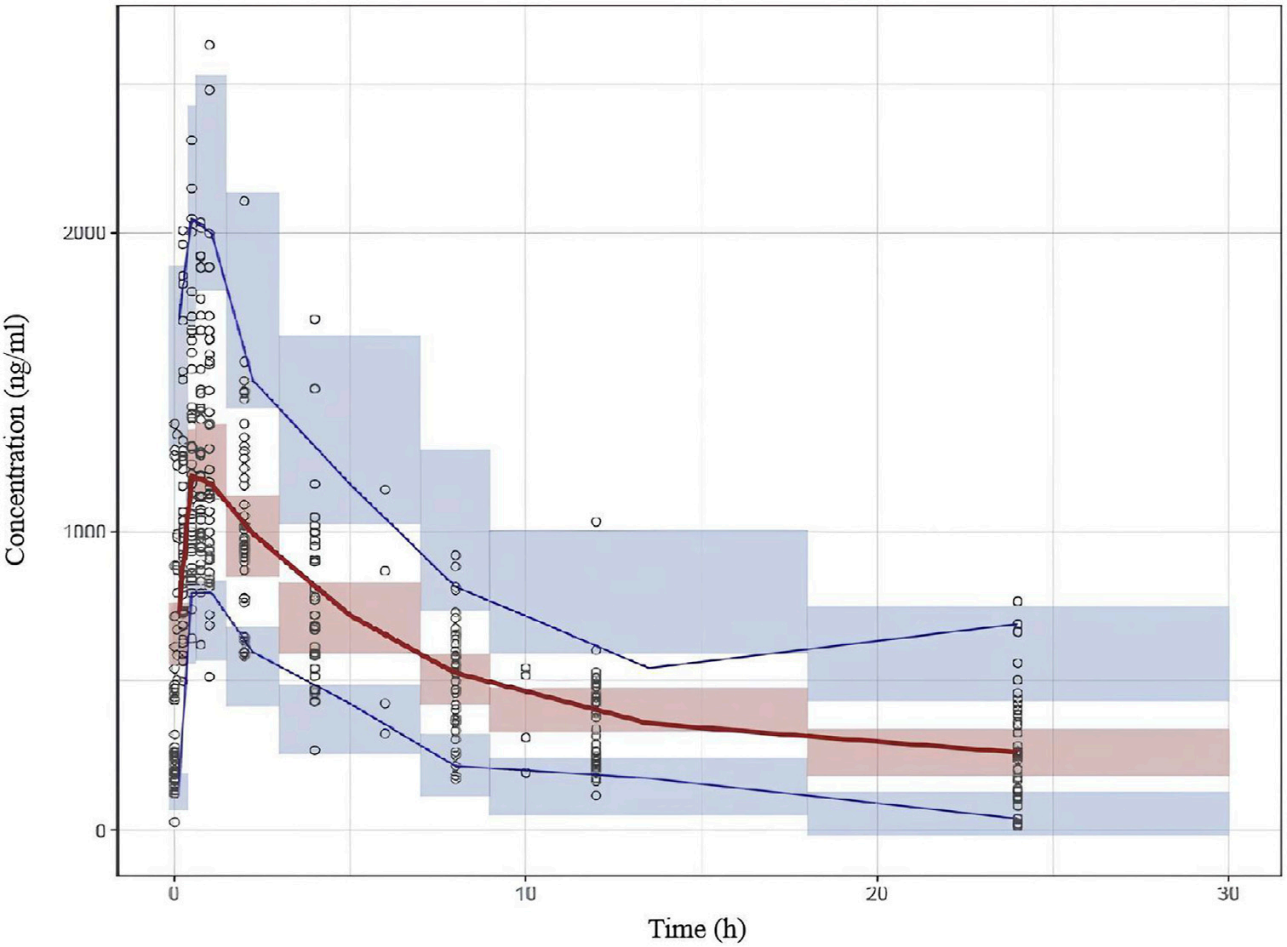
Visual predictive check (VPC) results of the final model. The hollow dots represent the observed data. Solid lines represent the 5th, 50th and 95th percentiles of observed data. Shaded areas represent the 95% confidence interval of the 5th, 50th and 95th percentiles of simulation.

**FIGURE 3:**
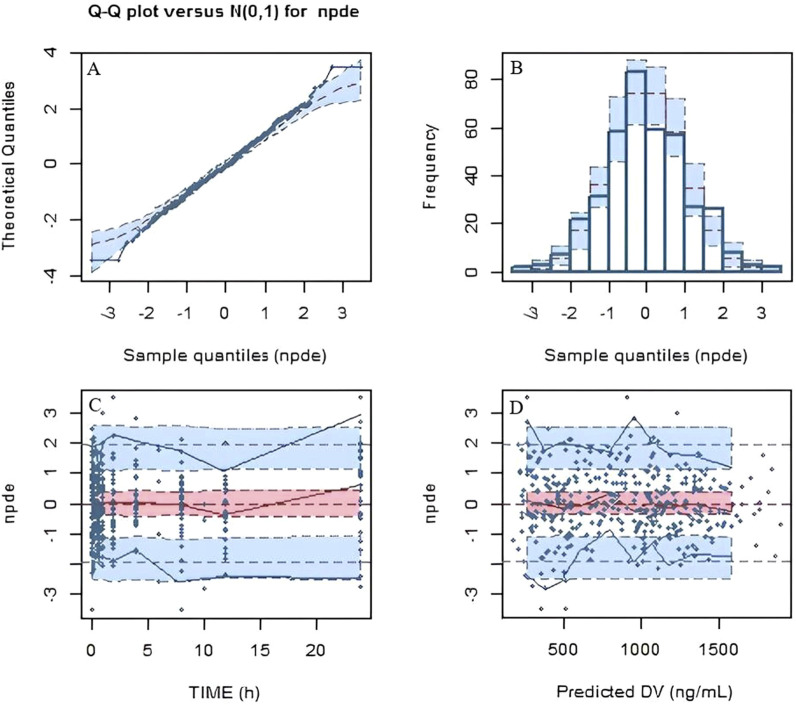
Normalized predictive distribution error (NPDE) of the final model. **(A)** Q-Q plot of the NPDE. **(B)** Histogram of the NPDE. **(C)** NPDE versus time after dose. **(D)** NPDE versus population predicted concentration.

Based on the final population pharmacokinetic parameters and the inter-individual variability, 1,000 simulations were conducted, and the VPC results are shown in Figure 2. It can be seen that the prediction intervals cover the 2.5th, 50th, and 97.5th percentiles of the observed values, indicating that the established model parameters are accurate and predictable. The results of the NPDE verification are shown in Figure 3, which show that the NPDE fits a normal distribution, and the statistical test *P* value is greater than 0.5.

For external validation, we conducted regression analysis using the population values of ka for three different concentrations of ropivacaine as calculated by the final model. The regression equation was derived as ka = 16.25ln (Conc)+9.1, with an *R* ^2^ value of 0.9911. Conc represents the concentration of ropivacaine injections. The equation indicates that for the concentration of 0.375 of ropivacaine, the value of ka is 25.05. The prediction error of the final model is shown in Table 4. The mean prediction error (MPE) and mean absolute prediction error (MAE) were −0.18% and 9.42%.

**TABLE 4:** Prediction error of the final model for patients with 0.375% ropivacaine.

ID	Time (h)	Observed concentration (ng/mL)	Predicted concentration (ng/mL)	Prediction error (%)
1	0.02	302.30	327.83	8.45%
1	0.25	1,205.40	1,098.80	−8.84%
1	0.50	1,230.50	1,282.20	4.20%
1	0.75	1,255.80	1,308.20	4.17%
1	1.00	1,437.60	1,222.00	−15.00%
1	2.00	781.90	946.14	21.01%
1	4.00	677.90	618.48	−8.77%
1	8.00	317.00	366.30	15.55%
1	12.00	315.30	282.87	−10.29%
1	24.00	174.40	183.23	5.06%
2	0.25	906.30	867.00	−4.34%
2	0.50	1,150.00	1,199.50	4.30%
2	0.75	1,295.20	1,318.70	1.81%
2	1.00	1,506.10	1,277.60	−15.17%
2	2.00	1,002.50	1,130.20	12.74%
2	6.00	830.50	746.32	−10.14%
2	12.00	470.30	498.00	5.89%
2	24.00	408.80	352.23	−13.84%

### Simulation

**FIGURE 4:**
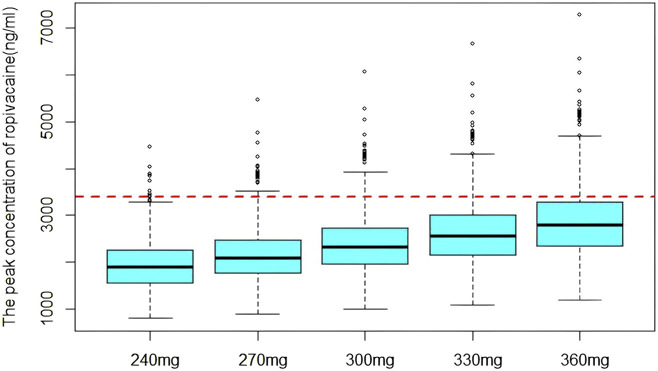
The distribution of simulated peak concentration of ropivacaine. The dashed line indicates the lower limit of concentration for toxic reaction.

The model results showed that the concentration of ropivacaine only affected ka, and there was no significant difference in other pharmacokinetic parameters. According to previous research, the utilization of 0.5% ropivacaine is generally recommended for performing serratus anterior plane block (Chen et al., 2020; Muhammad et al., 2024). The arterial drug concentrations in the anterior serratus plane block were simulated at doses of 4 mg/kg, 4.5 mg/kg, 5.0 mg/kg, 5.5 mg/kg, and 6 mg/kg with 0.5% ropivacaine in a 60 kg patients based on the final model. The distribution of the peak concentration of ropivacaine at different doses is shown in Figure 4. The proportion exceeding the lower limit of toxic reaction concentration (3400 ng/mL) was 1.2%, 2.2%, 5.3%, 11.8% and 20.6%, respectively.

## Discussion

The population pharmacokinetic studies reported in the literature mostly adopt a one-compartment model with first-order absorption for data analysis (Gromov et al., 2021; Matsota et al., 2024; Ollier et al., 2015). Riff et al. (2018) constructed a one-compartment population pharmacokinetic model for the treatment of pain after mastectomy for breast cancer with ropivacaine wound infiltration. The absorption model adopted the progressive absorption (Transit) model. In this study, multiple absorption models were employed to investigate the absorption process of ropivacaine in the serratus anterior plane block. Based on the model, fitting situation, and the objective function value (OFV), it was discovered that the goodness of fit of the zero-order and first-order mixed absorption was significantly improved compared to other absorption processes, suggesting that the absorption of ropivacaine in the serratus anterior plane block involves a small portion of the zero-order process (27.4%), and the absorption process is complex, which might also be one of the reasons for its relatively large individual variations.

In this study, the clearance rate of ropivacaine for the serratus anterior plane block was estimated to be 7.48 L/h, which is consistent with the results of the population pharmacokinetic model of ropivacaine for patients with erector spinae plane block established by Schwenk et al. (2023). The first-order absorption rate constant is significantly higher than the values reported in the literature (Schwenk et al., 2023). This might be attributed to the differences in the block location and the selection of the absorption model.

Moreover, concerning the influencing factors of ropivacaine pharmacokinetics, the covariate screening results of Schwenk et al. (2023) demonstrated that the patient’s height is a significant covariate for the absorption rate constant (ka). However, they only included 15 patients, with a relatively small amount of data, and the results require further validation. Matsota et al. (2024) established a population pharmacokinetic model of ropivacaine in arteriovenous blood after continuous thoracic paravertebral nerve block under ultrasound guidance, employing a one-compartment model with a pre-absorption compartment for the thoracic paravertebral space. Gender had a significant influence on CL/F, with the CL/F of females being lower than that of males. Moreover, there are also studies indicating that body weight and protein binding rate have an effect on the pharmacokinetics of ropivacaine (Aarons et al., 2011; Gromov et al., 2021). This study revealed that the concentration of ropivacaine injection significantly affected the first-order absorption rate constant. The first-order absorption rate constants of 0.25%, 0.5%, and 0.75% ropivacaine were 32.0, 19.4 and 14.4 h^−1^ respectively. That is, the peak time of low concentration ropivacaine was significantly shortened, which is consistent with the peak time calculated by our non-compartment model. Since serratus anterior plane block infuses the drug into the fascial space between the serratus anterior and latissimus dorsi muscles, a drug storage pump is formed, and the drug diffuses into the area to be operated. However, the concentration of 0.75% ropivacaine was relatively high, and the diffusion effect was not as good as that of the low concentration group, so the peak time was prolonged, and the T_max_ was greater than that of the 0.5% group and the 0.25% group. Therefore, low concentrations of ropivacaine have a rapid onset of anesthesia in clinical practice.

As the dosage used in the modeling data ranged from 150 to 243 mg, while the peak concentration ranged from 642.8 ng/mL to 2,630.3 ng/mL, which is less than the lower limit of toxic reaction concentration (3,400 ng/mL). None of the patients showed signs of local anesthetic systemic toxicity. Local anesthetic systemic toxicity can occur over the injection of local anesthetics after their passage through the blood. Clinical presentation of local anesthetic systemic toxicity consists of prodromal symptoms such as metallic taste, tinnitus, disorientation, logorrhea and dizziness, followed by seizures. At higher plasma drug concentrations, neurological depression and cardiac toxicity can occur (Riff et al., 2022). It reported that the risk factors of local anesthetic systemic toxicity included pregnancy, overweight, hepatic dysfunction, metabolic disease, and age >60 years (Macfarlane et al., 2021; Riff et al., 2022). The absence of adverse effects may be partially attributed to the relatively low proportion of enrolled patients who possessed these risk factors. In addition, the absorption of anesthetics and the plasma concentration achieved depends, among other factors, on the block site and its vascularization, the injection speed and the total dose administered (Zaballos et al., 2023). The blockade site selected in this study was the superficial layer of the serratus anterior muscle, which demonstrates a more stable and sustained effect (Qiu et al., 2021). Compared to the serratus anterior deep plane block, the absorption rate was markedly reduced and the peak concentration was lower (He et al., 2023). This further suggests that the shallow plane block technique is associated with enhanced safety. As the maximal blood concentration could be a relevant tool in confirming the diagnosis of local anesthetic systemic toxicity. We can using this population pharmacokinetic model and Bayesian estimation method to estimation the individual pharmacokinetic parameters and maximal concentration of ropivacaine, thereby facilitating clinical decision-making (Riff et al., 2022).

Simulation serves as a critical tool in pharmacokinetic modelling. By altering model parameters, covariates or dosing regimens, pharmacokinetic behavior under different scenarios can be simulated to support optimization therapeutic regimens. The simulation results demonstrated that when the total dose of 300 mg (5 mg/kg, 60 kg), the likelihood of peak concentration surpassing the lower limit of toxic concentration would exceed 5%. Therefore, when the superficial serratus anterior plane block is used in the clinic, the total dose of ropivacaine is recommended not to exceed 300 mg to avoid the occurrence of adverse reactions. It is worth mentioning that a meta-analysis report has shown that the concentration of ropivacaine over 2.2 μg/mL is thought to cause anesthetics poisoning. Therefore, in order to reduce the risk of dangerously high plasma levels, following maximum recommended dosage guidelines is necessary. Maximal doses are currently recommended according to the injection site (3 mg/kg in upper limb blocks and 4 mg/kg in lower limb blocks (Neal et al., 2012).

Our research does have a few limitations. First, the small sample size and narrow distribution of body size covariates might have contributed to the inability to detect covariate effects on the pharmacokinetic parameters of ropivacaine. Second, determination and evaluation of free drug concentrations were not performed in this study. Free plasma concentration of local anesthetics may be more important than total levels in predicting toxicity. Torup et al have reported that although the total plasma concentration of ropivacaine of ∼30% in patients exceeded the alert of neurotoxicity after bilateral transverse fascia block, no toxicity reaction occurred clinically, as the free plasma concentration was below the warning level (Torup et al., 2012). Finally, the accuracy of our model needs to be further verified. Therefore, it is necessary to further conduct multi-center, large sample randomized controlled clinical trials to further validate and enhance the reliability and applicability of the findings.

In conclusion, this study investigated the population pharmacokinetic characteristics of ropivacaine for the serratus anterior plane block and estimated the population pharmacokinetic parameters. It can offer a reference for the clinical rational administration of ropivacaine for the serratus anterior plane block in patients undergoing thoracoscopic lobectomy and enhance the quality and safety of anesthesia.

## Data Availability

The raw data supporting the conclusion of this article will be made available by the authors, without undue reservation.
